# Correlation analysis of clinical efficacy and prognosis of endoscopic submucosal dissection of early gastric cancer with peripheral blood indexes

**DOI:** 10.3389/fonc.2026.1615507

**Published:** 2026-03-06

**Authors:** Xiaojie Ying, Yuan Huang, Ling Zhang, Jianwei Hu, Bin Zhao

**Affiliations:** 1 Department of General Surgery, Shanghai Second People's Hospital (Shanghai Huangpu District Central Hospital), Shanghai, China; 2 Endoscopy Center and Endoscopy Research Institute, Shanghai Collaborative Innovation Center of Endoscopy, Zhongshan Hospital, Fudan University, Shanghai, China; 3 Surgical Department of TCM, Shanghai Second People's Hospital (Shanghai Huangpu District Central Hospital), Shanghai, China

**Keywords:** clinical efficacy, early gastric cancer, endoscopic submucosal dissection, peripheral blood indicators, prognosis

## Abstract

**Background:**

This study aimed to investigate the correlation between peripheral blood indicators and the clinical efficacy and prognosis of patients with early gastric cancer (EGC) undergoing endoscopic submucosal dissection (ESD).

**Methods:**

A single-center, retrospective cohort study was conducted on 120 EGC patients treated with ESD between January 2017 and August 2019. Patients were stratified into good (n=84) and poor (n=36) prognosis groups based on defined criteria. Univariate and multivariate logistic regression analyses were performed to identify factors influencing prognosis. The predictive performance of peripheral blood indicators was evaluated using receiver operating characteristic (ROC) curve analysis. Changes in biomarkers and patient-reported quality of life (assessed by QLQ-STO22) before and after ESD were compared, and their correlations were analyzed.

**Results:**

Univariate analysis revealed differences in Carbohydrate Antigen 19-9 (CA19-9), Carcinoembryonic Antigen (CEA), Neutrophil-to-lymphocyte ratio (NLR), Red blood cell distribution width (RDW), and Homocysteine (Hcy) levels between prognosis groups (all P<0.05). Multivariate analysis identified these five indicators as independent prognostic factors. ROC analysis demonstrated area under the curve (AUC) values of 0.723, 0.669, 0.757, 0.709, and 0.606 for CA19-9, CEA, NLR, RDW, and Hcy, respectively, in predicting prognosis. The combined model achieved an AUC of 0.950. Postoperative levels of all five biomarkers and QLQ-STO22 scores were lower than preoperative levels (all P<0.001). Furthermore, CA19-9, CEA, NLR, RDW, and Hcy showed positive correlations with QLQ-STO22 scores (all P<0.001).

**Conclusion:**

Peripheral blood indicators, particularly CA19-9, CEA, NLR, RDW, and Hcy, hold predictive value for the prognosis of EGC patients after ESD. Their postoperative reduction and positive correlation with quality-of-life scores suggest they reflect clinical efficacy. The combined use of these indicators provides the highest prognostic accuracy.

## Introduction

Early gastric cancer refers to gastric cancer confined to the superficial layer of the gastric wall (the first mucus layer or the second mucus layer) (1). At this stage, the lesion is shallow in scope and size, and there is usually no or only limited local lymph node metastasis. Patients may have no or only slight gastric discomfort and are difficult to detect (2). Its common symptoms are not specific, such as abdominal pain, abdominal distension, loss of appetite, and indigestion, and are easily confused with other gastric diseases (3). Therefore, when these symptoms occur, especially if they persist or worsen gradually, timely medical attention and further examination should be sought. China is a high-incidence country for gastric cancer, with a proportion of new cases and deaths from gastric cancer worldwide each year. Despite the rapid development of endoscopic diagnostic and therapeutic techniques in recent years, the mortality rate of gastric cancer remains high (4).

ESD is a minimally invasive technique that involves the complete dissection of the lesion from the submucosal layer using an endoscope. It is mainly used for the diagnosis and treatment of early gastrointestinal tumors (5, 6). While ESD has become a standard treatment for EGC, optimal post-procedural risk stratification remains challenging, particularly regarding the prediction of recurrence, metachronous lesions, and quality of life outcomes. This technique originated in Japan in the late 1990s and has since been widely applied in clinical practice (7). The primary objective of ESD is the diagnosis and treatment of early gastrointestinal tumors, offering the advantage of complete removal of superficial lesions in a single procedure (8). This surgical method is particularly suitable for the diagnosis and treatment of early esophageal cancer, early gastric cancer, gastrointestinal stromal tumors, and early colon tumors.

However, ESD also requires high technical proficiency and involves a challenging procedure (9). While the prognostic value of individual markers like CEA, CA19-9, NLR, RDW, and Hcy has been explored in the context of advanced gastric cancer or surgical resection (10), their specific role and combined utility in predicting outcomes for EGC patients treated with ESD remain less clearly defined. ESD represents a distinct therapeutic modality with unique oncological outcomes and follow-up considerations. After ESD, the primary clinical challenges are the risk of metachronous lesions and accurate assessment of treatment efficacy. Therefore, identifying accessible and reliable prognostic tools is paramount for risk stratification and personalized post-ESD surveillance. This study aims to bridge this gap by investigating the correlation between a panel of readily available peripheral blood indicators—encompassing tumor markers (CA19-9, CEA), inflammatory indices (NLR, RDW), and a metabolic marker (Hcy)—and the clinical efficacy and prognosis of ESD in patients with EGC.

Although individual peripheral blood markers such as CEA, CA19-9, NLR, RDW, and Hcy have been studied in the context of advanced gastric cancer or surgical resection (10), their integrated prognostic utility specifically in EGC patients treated with ESD—a distinct therapeutic modality with unique follow-up considerations—is not well established. Furthermore, the correlation between these readily available biomarkers and patient-reported outcomes (e.g., quality of life) after ESD has been underexplored. This study aims to address these gaps by evaluating a panel of peripheral blood indicators encompassing tumor markers (CA19-9, CEA), inflammatory indices (NLR, RDW), and a metabolic marker (Hcy) in relation to both clinical prognosis and patient-reported quality of life (assessed via QLQ-STO22) following ESD for EGC. We hypothesize that this multi-parameter biomarker profile can serve as a practical and comprehensive predictor for post-ESD outcomes, thereby informing personalized surveillance strategies. The novelty of this study lies in three aspects: Firstly, it specifically evaluates the prognostic utility of these biomarkers in the context of ESD, a minimally invasive treatment modality with distinct oncological outcomes, where validated prognostic tools are still needed. Secondly, it extends beyond the limitations of single-marker analysis by integrating multiple biological axes (tumor burden, systemic inflammation, metabolic stress) into a comprehensive risk assessment model. Thirdly, it offers a unique correlation between objective laboratory parameters and patient-reported quality of life (QoL) using the validated QLQ-STO22 questionnaire. This provides a more holistic and patient-centered evaluation of treatment efficacy, an approach that is scarcely explored in the existing literature on EGC post-ESD management.

## Methods

### Study design and population

**Figure 1:**
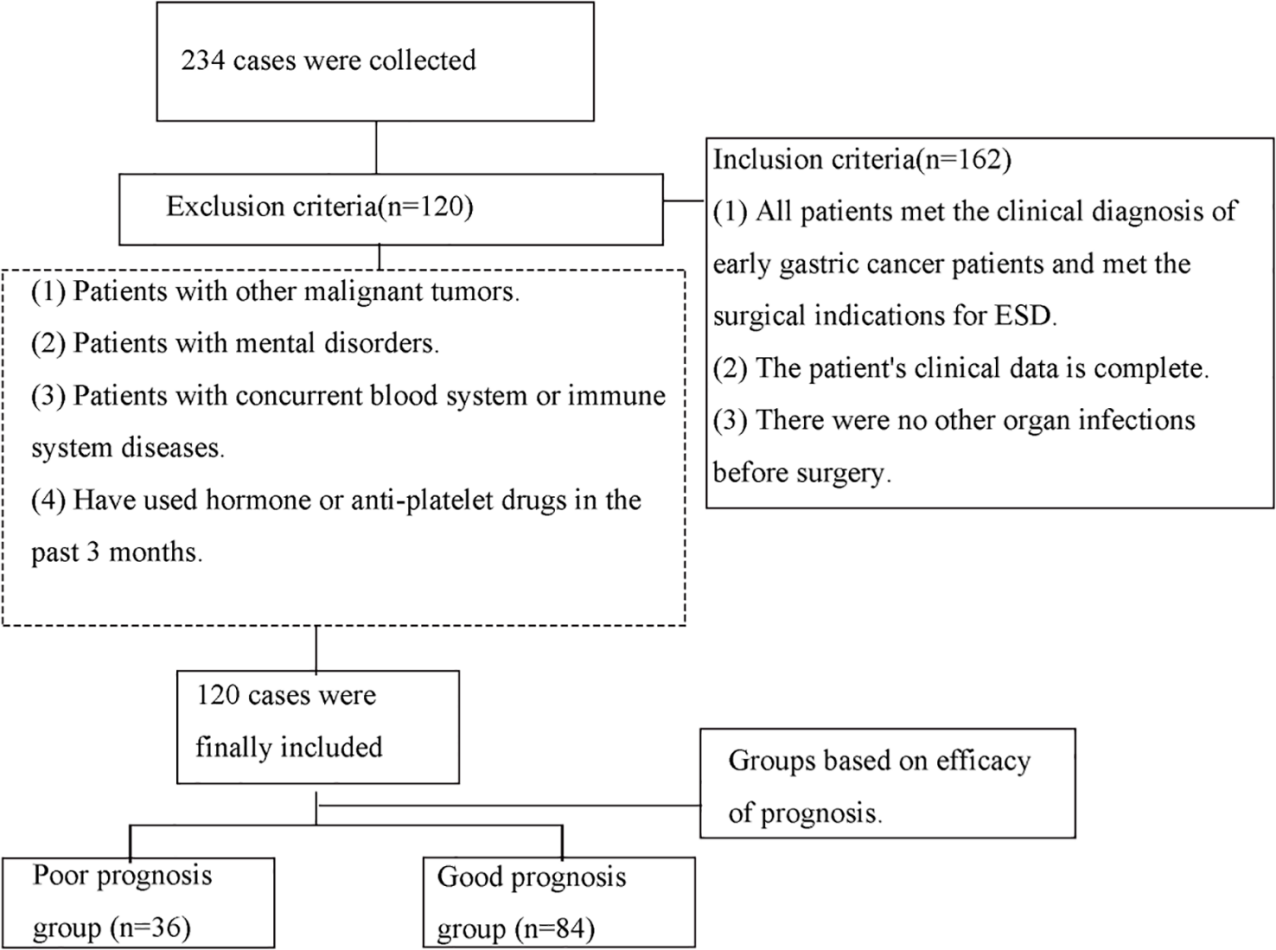
Flowchart of patients included.

This was a single-center, retrospective cohort study. A total of 234 patients were retrospectively included for screening and final 120 patients with early gastric cancer admitted to our hospital from January 2017 to August 2019. were retrospectively selected (**Figure 1**). Inclusion criteria: (1) All patients met the clinical diagnosis of early gastric cancer patients and met the surgical indications for ESD. (2) The patient’s clinical data is complete. (3) There were no other organ infections before surgery. Exclusion criteria: (1) Patients with other malignant tumors. (2) Patients with mental disorders. (3) Patients with concurrent blood system or immune system diseases. (4) Have used hormone or anti-platelet drugs in the past 3 months. This study was approved by the medical ethics committee of the Zhongshan Hospital, Fudan University. All patients signed informed consent forms.

### Data collection

General data of patients were collected using the electronic medical record system, and patients were divided into good prognosis group (n=84) and poor prognosis group (n=36) based on their prognosis. Analyze the correlation between patient efficacy and peripheral blood indicators. All patients completed follow-up, with a median follow-up duration of 4.1 years (range: 2–6 years) No patients were lost to follow-up or censored during the study period.

#### Prognosis criteria

The prognosis judgment time is the end of the follow-up time. Good prognosis: all target lesions disappeared completely, and non-target lesions did not progress or new lesions appeared. Poor prognosis: Patients who do not meet the above good conditions and/or meet the following conditions are considered to have a poor prognosis. (1) Patients with complications such as bleeding, perforation, and aspiration pneumonia (2) American Society of Anesthesiologists Physical Status ≤2 (11)(3) Patients with Prognostic Nutritional Index <50 (12, 13).

#### Efficacy assessment

Patient-reported outcomes were assessed using the gastric cancer-specific module (QLQ-STO22). The assessments were conducted at two time points: within one week before the ESD procedure (preoperative) and at the first scheduled postoperative outpatient review, which occurred between 2 to 6 months after ESD. To ensure consistency and data quality, all QLQ-STO22 assessments were administered in a standardized manner by two trained research nurses who were blinded to the patients’ laboratory results and prognostic grouping. The questionnaire was completed by patients independently in a quiet clinic room. If patients had difficulties (e.g., due to vision or literacy issues), the research nurses would read the questions aloud verbatim without providing any interpretation. Upon completion, the nurses immediately checked the questionnaires for any missing items and requested patients to complete them. The QLQ-STO22 scale includes five dimensions: dysphagia (DG), pain (PA), reflux (Rflx), eating restriction (EatR), and anxiety (Anx); there are 22 items scored from 1 to 4 points each. A higher score indicates more severe symptoms and a poorer quality of life.

### Statistical analysis

The collected experimental data were analyzed with SPSS 27.0 (SPSS Inc., Chicago, IL, USA). The normality of continuous data was assessed using the Shapiro-Wilk test. Count data were expressed as [n (%)] and analyzed using the chi-square test or Fisher’s exact test, as appropriate. Normally distributed continuous data were expressed as mean ± standard deviation (SD) and compared between the two prognosis groups using the independent samples t-test. Non-normally distributed data would be presented as median (interquartile range) and compared using the Mann-Whitney U test.

To address potential concerns regarding statistical power due to the sample size and group imbalance, a *post-hoc* power analysis was performed using G*Power (version 3.1.9.7). With the observed effect sizes for the primary biomarkers (NLR: d ≈ 1.06; CEA: d ≈ 2.96), α = 0.05, and the present sample sizes (n=84 good prognosis, n=36 poor prognosis), the achieved statistical power exceeded 0.95 for detecting differences in these key variables, suggesting that the study was adequately powered for the primary analyses despite the group imbalance. Furthermore, to mitigate the impact of group imbalance on logistic regression, we ensured that the number of events per variable (EPV) exceeded 10, meeting the recommended threshold for stable multivariable modeling.

Univariate analysis was first performed to identify potential factors associated with prognosis. Variables with a significance level of P < 0.05 in the univariate analysis were then included as independent variables in a multivariable binary logistic regression model (Enter method) to identify independent factors influencing the prognosis (dependent variable: poor prognosis=1, good prognosis=0). The results of the logistic regression are presented as odds ratios (ORs) with 95% confidence intervals (CIs). Given that multiple peripheral variables were compared between prognosis groups, the issue of multiple comparisons was addressed. For the univariate analysis, the False Discovery Rate (FDR) correction method (Benjamini-Hochberg procedure) was applied. Variables that remained after FDR correction (adjusted P < 0.05) were considered for inclusion in the subsequent multivariable logistic regression model.

The predictive performance of the identified independent peripheral blood indicators for prognosis was evaluated using Receiver Operating Characteristic (ROC) curve analysis. The area under the curve (AUC) was calculated, and the Youden’s index (Sensitivity + Specificity - 1) was used to determine the optimal cutoff value for each marker. The DeLong test was used to compare the AUCs of different indicators.

Changes in peripheral blood indicators and QLQ-STO22 scores before and after surgery were compared using the paired samples t-test. The correlation between the peripheral blood indicators and the QLQ-STO22 scores was assessed using Pearson correlation analysis. A two-sided P value < 0.05 was considered statistically for all tests.

## Results

### Univariate analysis of factors influencing the prognosis of early gastric cancer patients treated with ESD

There were no statistically differences (*P*>0.05) between the two groups of patients in terms of age, gender, alcohol history, hypertension, diabetes, disease duration, and HDL levels. However, there were statistically differences (*P* < 0.05) between the two groups in terms of CA19-9, CEA, NLR, RDW, and Hcy levels (**Table 1**). It was noteworthy that the mean values of the conventional tumor markers CA19–9 and CEA in both prognosis groups were found to be within the standard normal reference ranges(CEA < 5 μg/L, CA19-9 < 37 kU/L). This was consistent with the known characteristics of early gastric cancer, where elevation of these markers was uncommon. However, the statistically, albeit subtle, differences in these parameters between the good and poor prognosis groups suggested that even minor variations within the normal range may harbor prognostic significance.

**Table 1:** Univariate analysis of factors influencing the prognosis of early gastric cancer patients treated with ESD.

Characteristics	Good prognosis group (n=84)	Poor prognosis group (n=36)	t/χ^2^	*P value*
Age (years)	47.74 ± 2.54	47.60 ± 3.18	0.256	0.798
Gender, n (%)			0.014	0.905
Male	43(51.19)	18(50.00)		
Female	41(48.81)	18(50.00)		
Alcohol History, n (%)			0.225	0.635
Yes	27(32.14)	10(27.78)		
No	57(67.86)	26(72.22)		
Hypertension, n (%)				
Yes	21(25.00)	8(22.22)	0.106	0.745
No	63(75.00)	28(77.78)		
Diabetes, n (%)				
Yes	16(19.05)	5(13.89)	0.465	0.496
No	68(80.95)	31(86.11)		
Disease Duration (months)	2.70 ± 1.29	2.47 ± 1.34	0.885	0.378
CA19-9 (kU/L)	5.82 ± 1.26	6.73 ± 1.21	3.668	<0.001
CEA (μg/L)	1.48 ± 0.26	2.28 ± 0.27	15.270	0.000
HDL (mmol/L)	1.00 ± 0.09	0.98 ± 0.29	0.574	0.567
NLR (ratio)	2.07 ± 0.32	2.42 ± 0.33	5.449	<0.001
RDW (ratio)	12.94 ± 0.31	13.63 ± 0.30	11.280	<0.001
Hcy (μmol/L)	10.50 ± 0.33	11.71 ± 0.13	21.262	<0.001

### Multivariable logistic regression analysis of factors influencing the prognosis of early gastric cancer patients treated with ESD

The actual values of CA19-9, CEA, NLR, RDW and Hcy were assigned as independent variables, and the dependent variable for analysis was the prognosis (poor prognosis = 1, good prognosis = 0). The results of the multivariable logistic regression analysis showed that CA19-9, CEA, NLR, RDW, and Hcy were independent factors influencing the prognosis of early gastric cancer patients treated with ESD (*P* < 0.05) (**Table 2**). Collinearity diagnostics were performed for the five independent variables included in the logistic regression model. All variance inflation factor (VIF) values were below 1.2 (range: 1.036–1.106), and tolerance values were all above 0.9 (range: 0.904–0.965). These results confirm the absence of multicollinearity among the predictors, supporting the stability and reliability of the estimated regression coefficients.

**Table 2:** Multivariable logistic regression analysis of factors influencing the prognosis of early gastric cancer patients treated with ESD.

Risk factor	β value	SE value	Ward value	OR value	95% CI	*P value*	Tolerance	VIF
CA19-9 (kU/L)	0.584	0.197	8.785	1.793	1.219~2.638	<0.001	0.938	1.066
CEA (μg/L)	0.496	0.242	4.199	1.642	1.022~2.639	0.001	0.965	1.036
NLR (ratio)	0.301	0.112	7.215	1.351	1.085~1.683	<0.001	0.904	1.106
RDW (ratio)	0.308	0.114	7.310	1.361	1.088~1.702	<0.001	0.932	1.073
Hcy (μmol/L)	0.501	0.169	8.802	1.651	1.185~2.299	<0.001	0.957	1.045

### Predictive value of peripheral blood indicators in predicting the prognosis of early gastric cancer patients treated with ESD through ROC analysis

**Figure 2:**
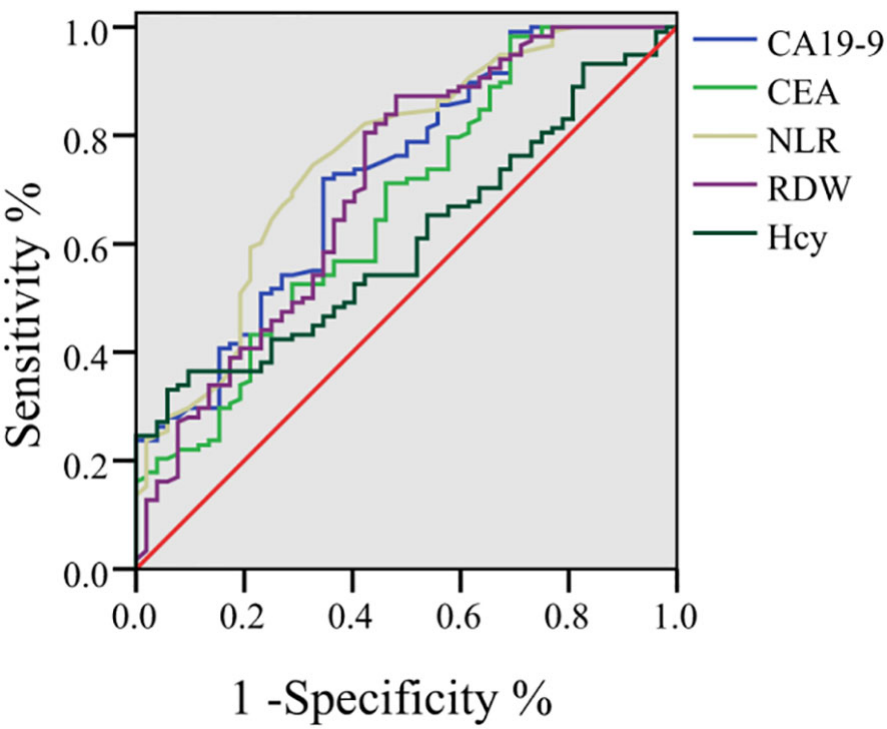
Predictive value of peripheral blood indicators in predicting the prognosis of early gastric cancer patients treated with ESD through ROC analysis.

ROC analysis showed that the area under the curve (AUC) for CA19–9 in predicting the prognosis was 0.723 (95% CI: 0.640–0.807), with a sensitivity of 72.00% and a specificity of 65.42%. For CEA, the AUC was 0.669 (95% CI: 0.579–0.759; sensitivity = 98.33%; specificity = 30.82%). The AUC for NLR was 0.757 (95% CI: 0.677–0.838; sensitivity = 74.63%; specificity = 67.33%). The AUC for RDW was 0.709 (95% CI: 0.620–0.798; sensitivity = 87.31%; specificity = 51.87%). The AUC for Hcy was 0.606 (95% CI: 0.520–0.692; sensitivity = 33.11%; specificity = 94.23%) (**Figure 2**).

**Figure 3:**
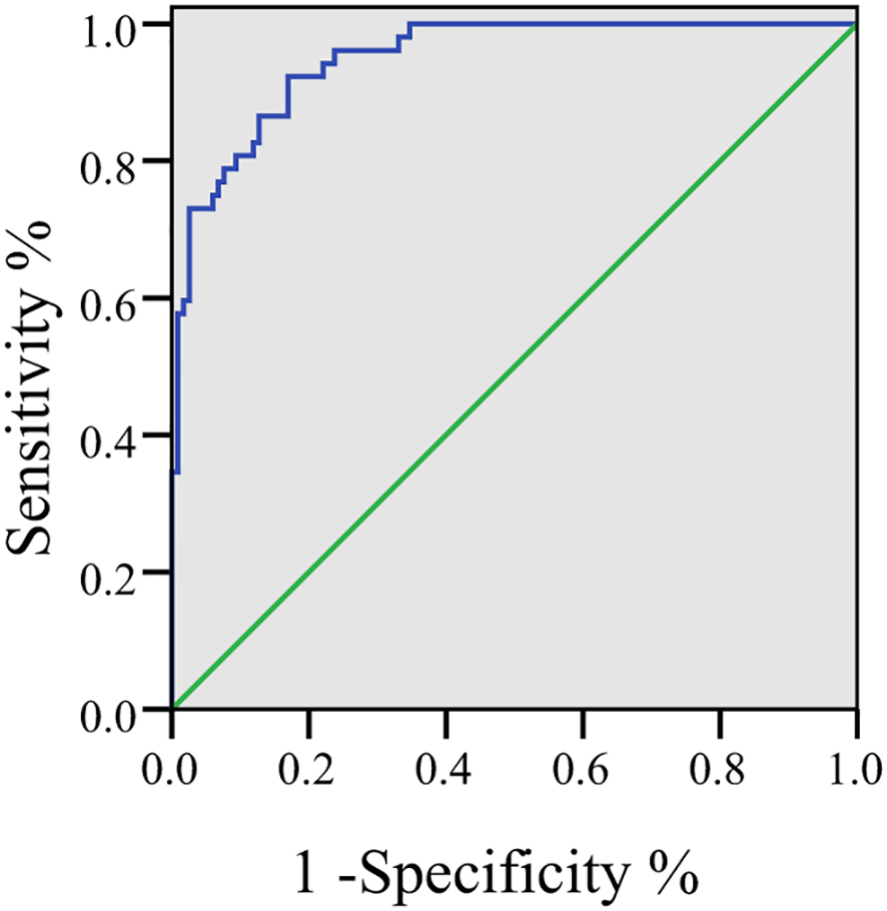
Roc curve for joint prediction of five indicators (CA19-9, CEA, NLR, RDW, Hcy).

To integrate the prognostic information, a combined predictive model was constructed based on the multivariable logistic regression coefficients. The AUC of this combined multi-marker model was 0.950 (95% CI: 0.920–0.980), which was higher than the AUC of any individual marker (all DeLong test P values < 0.001). At the optimal cut-off point (Youden’s index = 0.72), the combined model yielded a sensitivity of 91.54% and a specificity of 80.03% for predicting poor prognosis (**Figure 3**).

### Analysis of correlation between treatment efficacy and peripheral blood indicators

The levels of CA19-9, CEA, NLR, RDW, Hcy and QLQ-STO22 were lower after surgery compared to preoperative levels (*P* < 0.05). Although the absolute decreases in RDW and Hcy were relatively small (RDW: 13.67 to 12.88; Hcy: 11.67 to 10.46 µmol/L), the changes were statistically robust (*P* < 0.001). Both RDW and Hcy have narrow normal physiological ranges, and even minor shifts may reflect meaningful improvements in underlying systemic inflammation, oxidative stress, and metabolic homeostasis following tumor resection (**Table 3**). Pearson correlation analysis was conducted in order to assess the relationships between peripheral blood indicators and quality of life scores. A positive correlation was demonstrated between five biomarkers (CA19-9, CEA, NLR, RDW and Hcy) and the QLQ-STO22 score (all *P* < 0.001). The correlation coefficients (r) ranged from 0.690 to 0.776, indicating moderate to strong linear relationships. Furthermore, the coefficients of determination (R²) suggested that these peripheral blood indicators could explain approximately 47.6% to 60.2% of the variance in patient-reported quality of life scores (**Table 4**). This robustly confirms that elevated levels of these biomarkers are closely associated with worse symptomatic burden and poorer clinical efficacy.

**Table 3:** Changes in treatment efficacy and peripheral blood indicators before and after surgery.

Parameter	Preoperative (n=120)	Postoperative (n=120)	*t*	*P value*
CA19-9 (kU/L)	15.23 ± 2.54	5.94 ± 1.11	36.713	<0.001
CEA (μg/L)	4.36 ± 0.77	1.86 ± 0.21	34.313	<0.001
NLR (ratio)	2.34 ± 0.15	1.89 ± 0.14	24.025	<0.001
RDW (ratio)	13.67 ± 1.21	12.88 ± 1.13	5.227	<0.001
Hcy (μmol/L)	11.67 ± 1.24	10.46 ± 1.34	7.260	<0.001
QLQ-STO22	55.16 ± 6.67	35.46 ± 5.33	25.275	<0.001

**Table 4:** Correlation analysis of peripheral blood indicators with QLQ-STO22.

Parameter	QLQ-STO22		
	r	R^2^	*P value*
CA19-9 (kU/L)	0.762	0.581	<0.001
CEA (μg/L)	0.754	0.569	<0.001
NLR (ratio)	0.776	0.602	<0.001
RDW (ratio)	0.690	0.476	<0.001
Hcy (μmol/L)	0.747	0.558	<0.001

## Discussion

The main finding of this study is that a combined predictive model of a set of routine peripheral blood indicators (CA19 - 9, CEA, NLR, RDW and HCy) can be used as an auxiliary reference for predicting the prognosis of patients with EGC after ED. While these biomarkers themselves are not novel, the clinical context of their application is. Our work systematically validates their prognostic power specifically in the setting of ESD, a minimally invasive treatment for which optimal post-procedural risk stratification is still evolving. This addresses a direct clinical need for simple, cost-effective tools to guide follow-up intensity and patient counseling. The novelty of our study is further enhanced by the integration of objective biomarker data with patient-reported outcomes. We demonstrated not only that these biomarkers decreased after successful ESD but also that their levels were positively correlated with the QLQ-STO22 score, a validated measure of disease-specific symptoms and quality of life. This robust correlation (with R² values up to 60.2%) indicates that improvements in the underlying biological state—reflected by the normalization of these blood parameters—are closely paralleled by tangible improvements in how patients feel and function. This dual-confirmation strengthens the validity of using these peripheral blood indicators as a surrogate for comprehensive treatment efficacy assessment. In addition, NLR and RDW have been repeatedly proved to be valuable in the whole section of cancer (14, 15). Based on the above, this study decided to further analyze its relationship with early gastric cancer and provide reference for clinical practice.

The ROC analysis showed that CA19-9, CEA, NLR, RDW and Hcy were independent factors affecting the prognosis of patients with early gastric cancer treated by ESD. The reason may be that CEA is a commonly used tumor marker. The increase of CEA in patients with gastric cancer is usually related to the existence and development of the tumor. High levels of CEA may indicate a large tumor load or rapid disease progression, which affects the prognosis after ESD treatment. Previous studies by Shibata C et al. (16) have shown that CEA can be used as a predictor of postoperative recurrence of gastric cancer, similar to this prognosis-related view. The results of Gong X et al. (17) showed that anti-hp antibody combined with CA724, CA19–9 and CEA had important value in the identification of patients with early gastric cancer, which was similar to the results of this study. The results of this study showed that CA19-9, as a diagnostic index, was also associated with prognosis. In the future, it can be further analyzed. The increase of NLR usually reflects the enhancement of inflammatory response *in vivo*. Inflammatory response plays an important role in the occurrence and development of tumors, which may promote the progression and metastasis of tumors. Therefore, high NLR may be associated with poor prognosis. The decrease in RDW postoperatively may reflect an improvement in the underlying inflammatory state and nutritional status following the successful resection of the tumor (18, 19). As a marker associated with systemic inflammation and oxidative stress, the reduction in RDW aligns with the overall recovery trend observed after ESD treatment. High levels of Hcy are associated with an increased risk of a variety of chronic diseases, including cardiovascular disease and cancer, and may reflect oxidative stress and inflammatory responses in patients, thereby side-by-side showing the prognosis of patients. Further analysis, from the perspective of immune mechanism, lymphocytes play a key role in the anti-tumor immune response. If the anti-tumor cell immune response they rely on is reduced, it will weaken the body’s ability to recognize and kill tumor cells, which is not conducive to cancer prognosis (20). Neutrophils play a complex role in the development of tumors. On the one hand, they may participate in the formation of tumor inflammatory microenvironment and promote tumor angiogenesis and metastasis; on the other hand, their increase may reflect that the body is in a pro-inflammatory state, which is related to tumor progression. When tumors develop, increased neutrophil-and platelet-related inflammation, as well as reduced lymphocyte-dependent anti-tumor cell immune response, lead to increased neutrophil and platelet levels and decreased lymphocyte levels, which in turn increases NLR, suggesting a poor prognosis (21). In terms of RDW, RDW levels in patients with cancers such as colorectal cancer may be affected by inflammation and iron loss in chronic inflammatory states (21). Elevated inflammatory factors in cancer cells can impair erythropoietin activity, iron metabolism and homeostasis, leading to heterogeneous changes in red blood cell volume and increased RDW (21). Moreover, RDW is positively correlated with conventional tumor markers and pathological stages of many cancers, such as CEA and CA19–9 in colorectal cancer, CA50 and CA125 in gastric cancer (GC), etc., indicating that an increase in RDW may reflect tumor progression and poor prognosis (22, 23). Previous studies have also shown that high levels of NLR and PLR are associated with poor prognosis in colon cancer, ovarian cancer, and GC (21).

The pathophysiological rationale for this may lie in the biological processes these markers represent. CEA, for instance, is involved in cell adhesion, immune modulation, and metastasis. Even a slight upregulation within the ‘normal’ range could reflect a tumor phenotype with enhanced potential for invasion or interaction with the tumor microenvironment, such as epithelial-mesenchymal transition (EMT) (17, 18). Mild upregulation of CA19–9 may suggest that cancer cells have undergone subtle changes in their adhesion ability, making it easier for them to take root and grow in the gastric mucosa, creating conditions for further invasion. The slight upregulation of carcinoembryonic antigen not only reflects the enhanced phenotype and invasive potential of the tumor, but may also mean that immune regulation is unbalanced and the body’s ability to monitor and eliminate cancer cells is reduced. The slight increase in both suggests that early gastric cancer cells have certain biological activity. Although no obvious lesions have been formed, they have begun the process of progression to malignancy, providing key clues for early diagnosis and intervention. Therefore, in the context of EGC, low-level expression of these markers might be a surrogate for a more intrinsically aggressive biology, which becomes evident as a risk factor for unfavorable outcomes post-local resection. Furthermore, the prognostic strength of our model likely derives from the synergistic effect of combining markers reflecting different biological axes: tumor biology (CEA, CA19-9), systemic inflammation (NLR), and nutritional/metabolic stress (RDW, Hcy). This multi-parametric approach captures a more comprehensive picture of the host-tumor interaction than any single marker could. The systemic inflammatory state, indicated by NLR, is a well-known promoter of tumor progression and metastasis, and its presence, even in early-stage cancer, can portend a worse prognosis (19–22). The successful resolution of this inflammatory state and metabolic dysregulation after ESD, as evidenced by the postoperative decrease in all biomarkers, likely contributes to the improved clinical outcomes and quality of life observed in the good prognosis group.

These peripheral blood indexes and QLQ-STO22 decreased after operation, and were positively correlated with QLQ-STO22 score, which further confirmed its prediction and correlation with curative effect. It shows that peripheral blood indicators can show the clinical efficacy of patients, which is positively correlated with it. The reason is that CA19–9 and CEA are closely related to gastric cancer. The decrease of their levels directly reflects the reduction or elimination of tumor tissues after ESD treatment. After successful resection of tumor tissues, the concentration of these markers will naturally decrease, thus reflecting the effectiveness of treatment (24). As an index to evaluate the inflammatory response *in vivo*, the decrease of NLR means the reduction of inflammatory response. ESD treatment not only removes tumor tissue, but also may reduce the inflammatory response caused by tumor, thus improving the overall health status and quality of life of patients (25).The decrease of RDW may reflect the recovery of hematopoietic function and the improvement of red blood cell morphology. At the same time, the decrease of Hcy level may be related to the improvement of nutritional status and the recovery of metabolic function (26). These factors are important indicators to evaluate the therapeutic effect and prognosis, and their improvement further confirms the clinical efficacy of ESD treatment (27).

This study has several limitations that should be considered. First, its single-center, retrospective design inherently carries risks of selection bias. Second, the sample size, while sufficient for our initial analyses, is modest for developing a robust predictive model. The ultimate goal of identifying prognostic biomarkers is to guide clinical decision-making. While our study does not prospectively validate a clinical algorithm, the strength of the associations we observed allows us to propose a potential pathway for clinical translation. It is recommended that future research be conducted in the form of large-scale, multi-center, prospective studies. The purpose of such studies would be to independently validate the prognostic utility of risk score integration. The risk score integration in question would be that of the weighted values of CA19-9, CEA, NLR, RDW, and Hcy. This score could be used to stratify EGC patients immediately after ESD into distinct risk categories. For instance, patients classified as ‘low-risk’ by this score might be candidates for a less intensive follow-up regimen, thereby reducing the burden of endoscopic surveillance and associated healthcare costs. Conversely, ‘high-risk’ patients could be allocated to more vigilant follow-up protocols, including shorter-interval endoscopies and consideration of cross-sectional imaging, to facilitate the earliest possible detection of disease recurrence or metachronous lesions. The routine availability and low cost of these blood tests make such a personalized strategy highly feasible and potentially cost-effective. Our findings provide the necessary rationale and preliminary data to design such a prospective, interventionist study.

Furthermore, investigating the molecular mechanisms linking subtle changes in these peripheral markers to tumor biology in EGC could provide deeper insights. The strong positive correlations observed between the peripheral blood indicators and the QLQ-STO22 score at baseline are noteworthy. These high correlation coefficients suggest that the biological states reflected by these biomarkers—including systemic inflammation, tumor burden, and metabolic stress—are closely intertwined with the patient’s symptomatic experience prior to treatment. For instance, systemic inflammation (elevated NLR) can directly contribute to cancer-related symptoms like fatigue and pain, while a more aggressive lesion (potentially signaled by slightly higher CEA/CA19-9) could cause more eating restrictions or reflux. This alignment between objective laboratory data and subjective patient reports strengthens the biological plausibility of our findings. However, it is crucial to interpret these correlations with caution; they represent a strong association rather than causation, and the biomarkers are not intended to replace the critical assessment of patient-reported outcomes in clinical practice.

Based on the results of this study, the clinical strategy can be adjusted accordingly. Patients can be divided into low-risk groups and high-risk groups based on the risk stratification of these indicators after surgery. Low-risk patients can adopt a more relaxed follow-up plan to reduce the frequency of endoscopic review, so as to reduce medical costs and patient burden; high-risk patients need to receive closer monitoring, including shortening the endoscopic review interval and considering cross-sectional imaging, in order to detect disease recurrence or metachronous lesions as soon as possible. However, this study still has limitations, including possible selection bias introduced by retrospective design, possible bias in single-center data, and relatively limited sample size, all of which may affect the universality and accuracy of the results. The grouping criteria used in this study are not fully applicable to the prognosis of early gastric cancer, so they need to be comprehensively considered in clinical application as an auxiliary judgment criterion. Therefore, future research needs to use a multi-center, prospective cohort design to further verify the prognostic value of these peripheral blood indicators and explore the molecular biological mechanisms behind them, in order to better understand their association with tumor biological behavior. On this basis, a more accurate risk scoring system can be developed to integrate the weighted values of the above indicators to provide individualized follow-up management plans for patients after ESD. This not only helps optimize the allocation of medical resources, but also improves the long-term quality of life and prognosis of patients.

## Conclusion

In conclusion, peripheral blood indicators have indeed demonstrated high value in predicting the prognosis of patients with early gastric cancer treated with ESD. They can reflect the clinical efficacy of patients and help doctors more accurately predict the treatment outcomes and prognosis of patients, thereby enabling the development of more individualized treatment plans.

## Data Availability

The raw data supporting the conclusions of this article will be made available by the authors, without undue reservation.
